# Comment on; post-transcatheter aortic valve replacement acute kidney injury; prevention rather than cure

**DOI:** 10.15171/jrip.2017.02

**Published:** 2016-10-21

**Authors:** Macaulay Amechi Chukwukadibia Onuigbo, Nneoma Agbasi

**Affiliations:** ^1^Department of Nephrology, Mayo Clinic Health System, Eau Claire, WI 54702, USA; ^2^North East London NHS Foundation Trust, UK

**Keywords:** Transcatheter aortic valve, replacement, Surgical aortic valve, replacement, Minimally invasive, aortic valve replacement, Acute, kidney injury, Renal replacement, therapy, Hemodialysis

**Implication for health policy/practice/research/medical education:**

There is newly accumulating evidence in the surgery-acute kidney injury (AKI) literature that intraoperative hypotension (IOH) is a formidable and yet preventable causative factor in precipitating postoperative AKI. Postoperative AKI portends both increased short- and long-term morbidity and mortality, increased length of stay and higher healthcare costs. IOH is preventable. We present a case of AKI that led to the syndrome of rapid onset end stage renal disease (SORO-ESRD) in a 73-year-old diabetic hypertensive chronic kidney disease (CKD) male patient in 2012 following minimally invasive surgical aortic valve replacement. We call for more attention to be paid to IOH and to avoid too low blood pressures in the operating room. We posit that systolic blood pressure targets ≥ 90 mm Hg and/or MABP targets ≥ 60 mm Hg potentially would improve postoperative renal outcomes.

## Dear Editor,

Huber et al in a single-center cohort study of 3646 patients who underwent inpatient vascular surgery between 2000 and 2010 at a tertiary care teaching hospital demonstrated that perioperative acute kidney injury (AKI) occurred in 1801 (49.4%) patients (1). Besides, adjusted cardiovascular mortality estimates at 10 years were 17%, 31%, 30%, and 41%, for patients with no kidney disease, for patients with AKI but without chronic kidney disease (CKD), for patients with CKD but without AKI, and for patients with AKI and CKD, respectively (1). Until very recently, in the cognate AKI-surgery literature, intraoperative hypotension (IOH) following hypotensive anesthesia or controlled hypotension in the operating room has been accepted to not pose any significant short-term and/or long-term consequences on renal function (2). Nevertheless, in the last few years there has been an increasing awareness of the impact of IOH as a formidable albeit preventable factor in the causation of postoperative AKI (3-7).

AKI following aortic valve replacement (AVR) results in longer length of hospital stay, increased utilization of healthcare resources and at the same time is independently associated with both higher short- and long-term mortality (8,9). AKI complicating surgical AVR (SAVR) has been variously reported in the literature (10-13).The increasing trend towards less invasive surgical options for the management of symptomatic AVR especially in patients with multiple comorbidities has led to such innovative surgical techniques as minimally invasive AVR, and recently, transcatheter AVR (TAVR) (14-16).

Very lately, Cheungpasitporn and colleagues from Mayo Clinic, Rochester, in an extensive review of the TAVR literature published in this journal, *Journal of Renal Injury Prevention*, noted that TAVR has now emerged as a viable treatment option for high-risk patients with severe aortic stenosis (AS) who are not suitable candidates for SAVR (17). The authors acknowledged that despite encouraging published outcomes, AKI after TAVR is common and lowers the survival of patients after TAVR (17). Factors identified to contribute to AKI following TAVR include preoperative factors (older age, pre-existing chronic kidney disease, iodinated contrast exposure, congestive heart failure, peripheral vascular disease, diabetes mellitus), periprocedural factors (bleeding and blood transfusion, embolic events, contrast agents, hypotension from rapid ventricular pacing, complicated cases requiring intra-aortic balloon pump) and postoperative factors (vasoconstricting agents, nephrotoxins, decreased heart function, hemodynamic instability, grade of aortic regurgitation after the procedure)(10-13,17).

From a preventative standpoint, a critical scrutiny of the above contributory factors to postoperative AKI following TAVR reveals that most of the factors with the exception of IOH and nephrotoxic exposure are non-modifiable factors (6,7,18). IOH, albeit a very preventable causative factor, has for a long time been unrecognized and neglected as a very important contributing factor in precipitating postoperative AKI (6,7). Moreover, postoperative AKI must be recognized in its full perspective - it must be conceded that AKI, including post-operative AKI, does sometimes lead to acute yet irreversible end stage renal disease, a syndrome of rapid onset end stage renal disease (SORO-ESRD), that we first described in the journal “*Renal Failure*,” in 2010 (19).

Walsh et al analyzed 33330 non-cardiac surgeries at the Cleveland Clinic, Ohio and evaluated the association between intraoperative mean arterial pressure (MAP) from less than 55 to 75 mm Hg and postoperative AKI and myocardial injury to determine the threshold of MAP where risk is increased (4). AKI and myocardial injury developed in 2478 (7.4%) and 770 (2.3%) surgeries, respectively. The MAP threshold where the risk for both outcomes increased was less than 55 mm Hg. Compared with never developing a MAP less than 55 mm Hg, those with a MAP less than 55 mm Hg for 1-5, 6-10, 11-20, and more than 20minutes had graded increases in their risk of the two outcomes (AKI: 1.18 [95% CI, 1.06-1.31], 1.19 [1.03-1.39], 1.32 [1.11-1.56], and 1.51 [1.24-1.84], respectively; myocardial injury 1.30 [1.06-1.5], 1.47 [1.13-1.93], 1.79 [1.33-2.39], and 1.82 [1.31-2.55], respectively) (4). In a similar study, Sun et al, in a recent prospective cohort study of the association of IOH with AKI in 5127 patients undergoing non-cardiac surgery with invasive MAP monitoring and length of stay of one or more days in the hospital had established that AKI occurred in 324 (6.3%) patients and was associated with MAP <60 mm Hg for 11–20 minutes, and MAP <50 mm Hg for more than 10 minutes in a clearly graded fashion (5).

## Case Report

We present below a brief description of our experience in 2012 of a then 73-year old obese hypertensive diabetic CKD stage III Caucasian male patient seen at the Renal Unit of the Mayo Clinic Health System, Eau Claire, in Northwestern Wisconsin, USA, who developed acute yet irreversible AKI in March 2012 following a minimally invasive AVR for symptomatic AS and where IOH played a crucial instrumental role in precipitating AKI. The patient, now 77 years old, remains on maintenance in-center outpatient hemodialysis, three times a week, for ESRD. We would argue that whereas if IOH was prevented here, the patient plausibly would have only experienced mild AKI, would not have needed renal replacement therapy (RRT) and would not be on maintenance hemodialysis four long years later, in March 2016.

**Figure 1:**
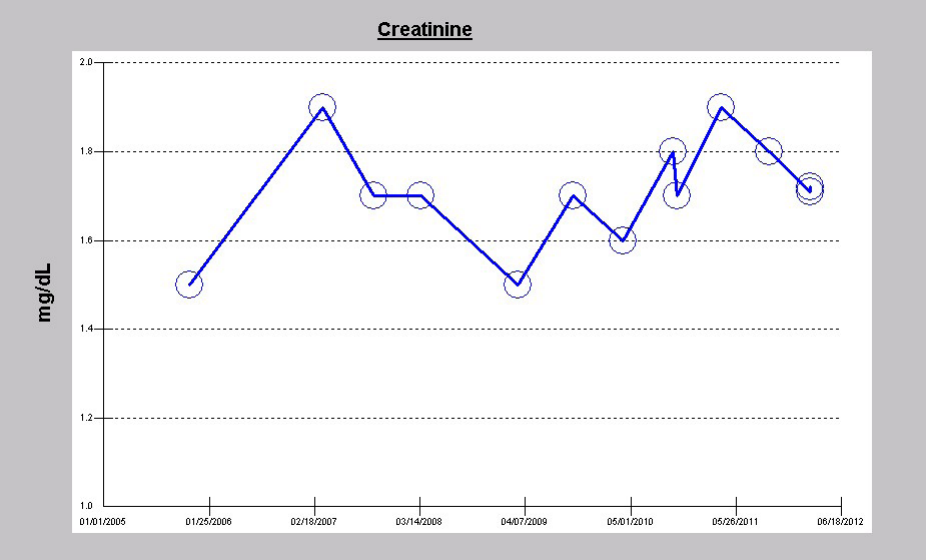
Stable serum creatinine of 1.5-1.9 mg/dL between November 2004 and February 2012.

A 73-year-old Caucasian man, nonsmoker with past medical history for hypertension, type II diabetes mellitus, obesity, sleep apnea, dyslipidemia, atrial fibrillation, osteoarthritis, a remote history of temporary need for hemodialysis in 2004 for AKI secondary to autoimmune hepatitis, with baseline serum creatinine of 1.5-1.90 mg/dL between November 2004 and February 2012 was evaluated for dyspnea in February 2012 (Figure 1). Outpatient medications included amlodipine 10 mg/d, atenolol-chlorthalidone 100/25 daily, atorvastatin 80 mg/d, insulin, lisinopril 40 mg/d and multivitamins with minerals daily. He was admitted to the CCU late in February 2012 with acutely worsening heart failure, severe dyspnea and chest pressure. Temperature 36.6, pulse 57 BPM, respiratory rate 35/min, BP 147/76 mm Hg, and pulse oximetry 93% on 3 liters nasal cannula. HEENT; he had bilateral basal inspiratory crackles and a 3/6 ejection systolic ejection murmur predominantly in the aortic region. Hemoglobin was 10.7 g/dL with otherwise normal CBC. Electrolytes were normal and serum creatinine was stable at 1.7 mg/dL. BNP was 1314 ρg/mL (0-100). Urinalysis was unremarkable, without dipstick proteinuria. His initial electrocardiogram (ECG) in the ED showed regular wide complex tachycardia of 120 BPM, in atrial flutter with left bundle branch block (LBBB). His chest x-ray showed pulmonary edema. The patient’s chest pressure resolved following intravenous Furosemide and nitroglycerin 0.1 mcg/kg/min infusion. He subsequently ruled out for acute Myocardial infarction (MI) by ECG and cardiac enzymes. Echocardiogram demonstrated a left ventricular ejection fraction (LVEF) of 50%, paradoxical septal motion consistent with LBBB, grade 2/4 diastolic dysfunction with increased filling pressures, and severe AS with mean gradient of 48 mm Hg, peak gradient of 73.6 mm Hg, peak velocity of 4.3 m/s, dimensionless index (LVOT/AV VTI ratio) of 0.25, and valve area of 1 cm^2^ (3-4 cm^2^, normal).

He underwent coronary angiogram which did not show significant coronary artery disease. He underwent prophylactic dental extraction for severe caries and subsequently on March 2, 2012 a minimally invasive surgical AVR with a 25 mm St. Jude Epic stented tissue valve was performed with temporary pacing wires, right femoral venous percutaneous cannulation for cardiopulmonary bypass.

**Figure 2:**
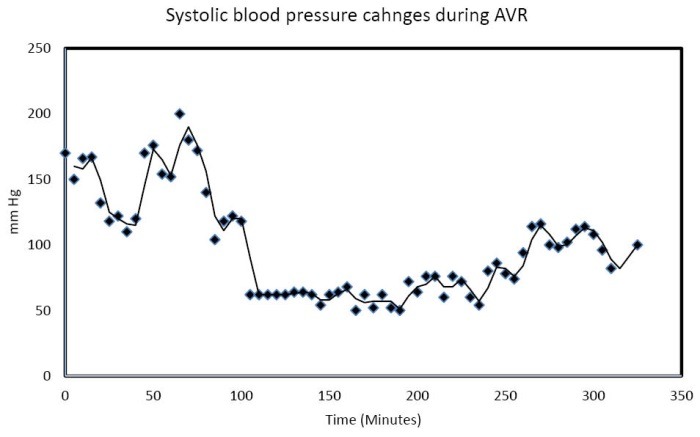
Systolic blood pressure trajectory during the minimally invasive AVR procedure in early March 2012.

**Figure 3:**
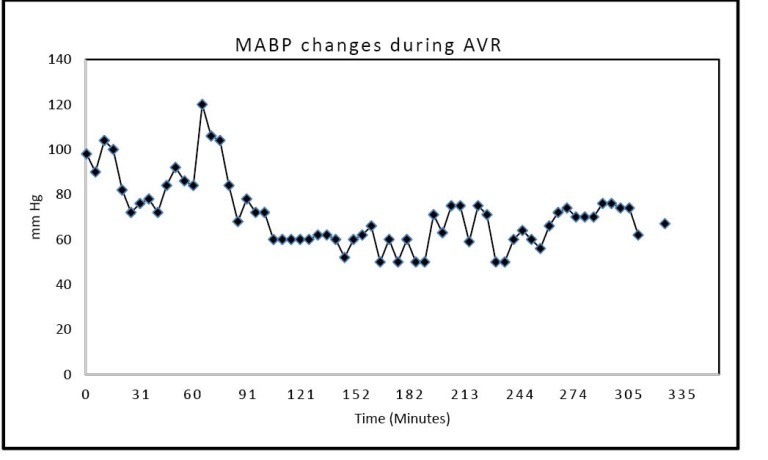
Mean arterial blood pressure trajectory during the minimally invasive AVR procedure in early March 2012.

The AVR procedure was carried out through an 8 cm right anterior chest mid-third intercostal space incision, made directly over the fourth rib. One unit of packed red blood cells was given. The postoperative transesophageal echocardiogram demonstrated an LV of 50% with a normal functioning aortic valve prosthesis. The bypass time was 150 minutes. The cross clamp time was 101 minutes. Intraoperative continuous blood pressure recordings during the nearly 8-hour long procedure demonstrated significant episodes of IOH, with systolic blood pressure values as low as 50 mm Hg, MAP as low as 50 mm Hg, and diastolic blood pressure values as low as 44 mm Hg repeatedly recorded during the operation (Figures 2 and 3).

**Figure 4:**
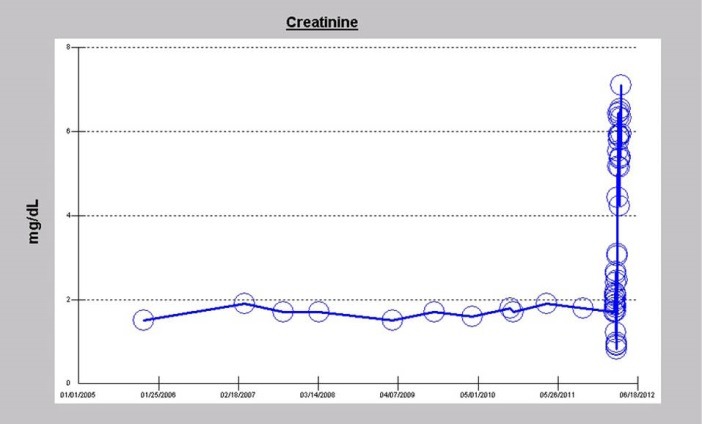
Serum creatinine trajectory soon following the minimally invasive AVR procedure in early March 2012.

**Figure 5:**
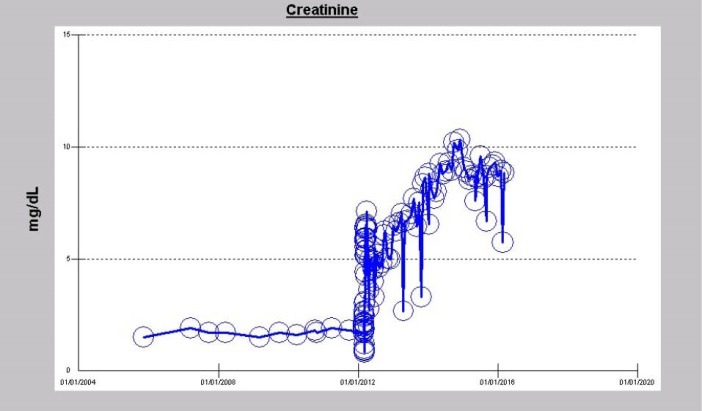
Serum creatinine trajectory from November 2004, immediately following the minimally invasive AVR procedure in early March 2012, through early March 2016

Because of post-operative AKI, progressive volume overload and oligoanuria despite high-dose continuous intravenous furosemide infusion, he needed RRT to be initiated within 24 hours of the AVR. He had been on chronic ACE inhibition for many years with Lisinopril 40 mg daily immediately prior to the AVR procedure; last dose of Lisinopril documented was on February 26, 2012 as an inpatient. Serum creatinine rose very quickly and has since remained elevated (Figures 4 and 5). His initial hemodialysis vascular access was a temporary right femoral vein dialysis catheter, on March 3, 2012. The right femoral dialysis catheter was subsequently replaced by a left internal jugular vein tunneled Palindrome dialysis catheter (Permcath) for dialysis access on March 15, 2012. Four months later, when it became clear that there was no renal recovery, his status was converted to ESRD, and a transposed cephalic vein AV fistula was created in the left upper arm on July 10, 2012. This was converted in January 2013 to a left upper arm loop graft die to poor maturation of the prior placed AVF. He has since then continued his hemodialysis treatments using this AVF graft to date. He remains an anuric ESRD patient, with a serum creatinine of 8.85 mg/dL as at March 2, 2016, exactly four years after the minimally invasive AVR procedure (Figure 5).

## Conclusion

Increasingly, there is accumulating evidence that IOH is a formidable and yet preventable causative factor in precipitating postoperative AKI. Postoperative AKI portends both increased short- and long-term mortality. AKI can indeed sometimes lead to acute yet irreversible and permanent renal failure, the syndrome of rapid onset end stage renal disease (SORO-ESRD). IOH is preventable. We strongly posit that every effort to eliminate IOH in the operating room is worth every effort (6,7,20). Randomized controlled trials to test for the most appropriate level of intraoperative blood pressures to prevent the precipitation of postoperative AKI are long overdue. Furthermore, from our anecdotal experiences here at the Mayo Clinic Health System in Northwestern Wisconsin, elective surgical procedures, both cardiac and non-cardiac, should be deferred until hypertension is controlled (6,7,20). As a result, the consequences of IOH on renal outcomes will be mitigated or at least minimized (6,7,20).

## Authors’ contribution

MACO; conception, design, acquisition of data, data analysis, interpretation of data, literature review, drafting the article and final approval of manuscript. NA; literature review, drafting the article and final approval of manuscript.

## Conflicts of interest

None.

## Ethical considerations

Ethical issues (including plagiarism, data fabrication, double publication) have been completely observed by the authors. Written consent was obtained from the patient for publication of the study.

## Funding/Support

None.
